# Stereogenic boron in 2-amino-1,1-diphenylethanol-based boronate–imine and amine complexes

**DOI:** 10.3762/bjoc.7.72

**Published:** 2011-05-16

**Authors:** Sebastian Schlecht, Walter Frank, Manfred Braun

**Affiliations:** 1 Institute of Organic and Macromolecular Chemistry, University of Düsseldorf, Universitätsstr. 1, D-40225 Düsseldorf, Germany; 2 Institute of Inorganic and Structural Chemistry, University of Düsseldorf, Universitätsstr. 1, D-40225 Düsseldorf, Germany

**Keywords:** boron, chirality, coordination chemistry, crystal structure, stereochemistry

## Abstract

Racemic boronate–imine and boronate–amine complexes **8** and **10**, both featuring a stereogenic boron atom were synthesized from 2-amino-1,1-diphenylethanol (**5**) and characterized by crystal structure analyses. Proof of enantiomerism at the boron center for the novel boronate–amine complex **10** was established by separation of the enantiomers. Racemization barriers were found to be in the same range for both amine and imine complexes (100–110 kJ/mol).

## Introduction

**Scheme 1:**
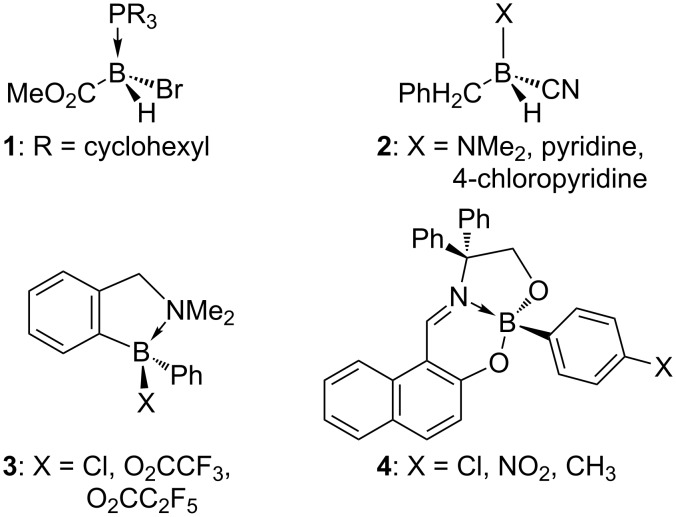
Stereogenic boron in resolved acyclic and cyclic complexes **1**–**4**.

Enantiomerism of main group hetero elements has been investigated thoroughly for compounds with stereogenic sulfur [1], phosphorus [2], nitrogen [3] and silicon atoms [4]. By comparison, stereogenic tetrahedral-coordinated boron has been studied to a much lesser extent. This is partly because it was incorporated in a chiral environment generated by enantiomerically pure ligands [5–9] or counter-ions [10], although some of these complexes have been obtained in a diastereoselective manner [6–9]. On the other hand, enantiomerism at boron has been observed in acyclic tetra-coordinated complexes **1** [11] and **2** [12], in acyloxyboranes **3** [13–16] with electron with-drawing substituents X (Scheme 1) that turned out to be crucial to the configurational stability at boron, and recently, in boronate complexes of boradiazaindacene [17]. Pursuing the concept of tridentate ligands derived from 2-amino-2,2-diphenylethanol we have been able to obtain boronate–imine complexes **4** with boron as a stable stereogenic center (Scheme 1). Their racemization barrier was measured and the absolute configuration of the isolated enantiomers was determined by a comparison of the measured and calculated CD spectra [18]. In this article, we describe the synthesis, resolution and characterization of chiral boronate–imine and boronate–amine complexes **8** and **10**, respectively. Their backbone is derived from the regioisomeric 2-amino-1,1-diphenylethanol (**5**) which features the geminal diphenyloxymethyl motif [19].

## Results and Discussion

**Scheme 2:**
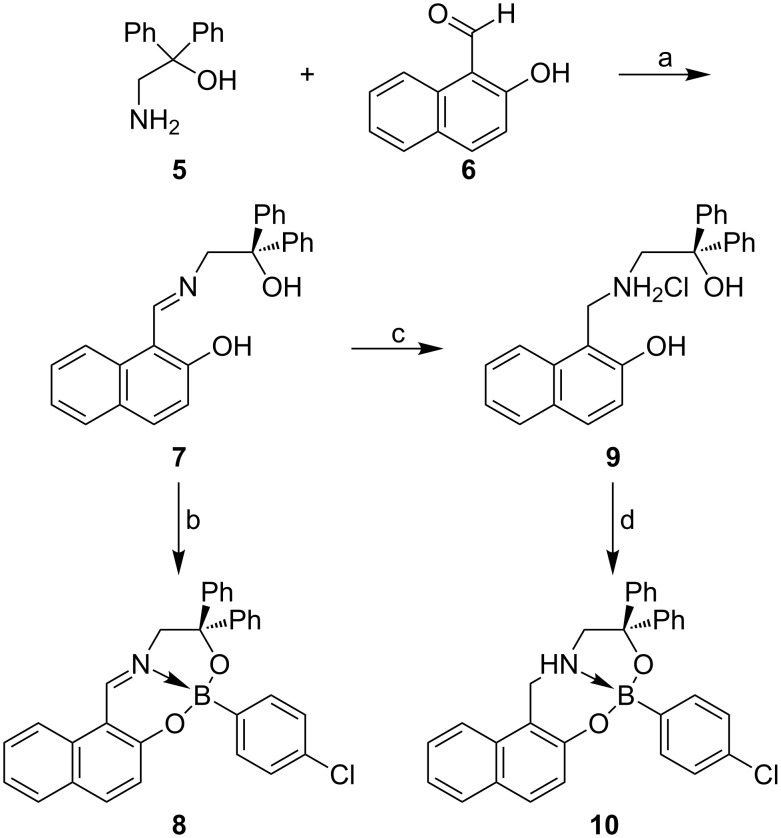
Synthesis of the racemic boronate–imine complex **8** and boronate–amine complex **10**. Reagents and conditions: a) Na_2_SO_4_, MeOH/THF (1:1), 18 h reflux, 87%; b) 4-chlorophenylboronic acid, molecular sieves 3Å, toluene, 20 h reflux, 17%; c) NaCNBH_3_, MeOH, hydrochloric acid, 25 °C, 0.5 h; d), 4-chlorophenylboronic acid, NaHCO_3_, toluene, 4 h reflux, 63%.

**Figure 1:**
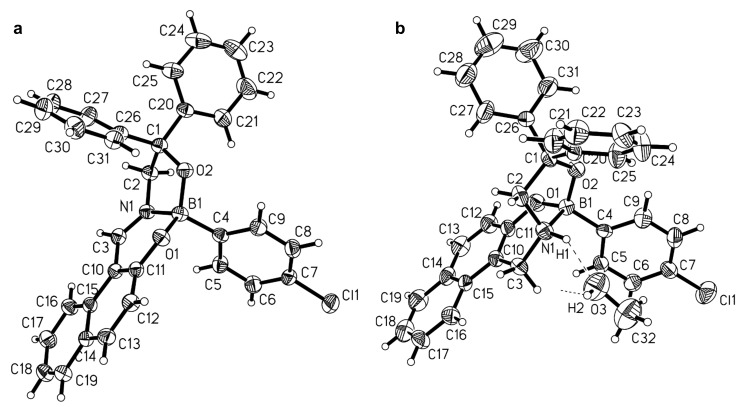
Solid-state structure of boronate–imine complex **8** (a) and the chosen asymmetric unit of the crystal structure of boronate–amine-complex solvate **10**·CH_3_OH (b) showing the hydrogen bonding of the methanol molecule (right). Displacement ellipsoids are drawn at the 30% probability level, radii of hydrogen atoms are chosen arbitrarily and the hydrogen atom labels are omitted for clarity. Selected bond distances and angles are given in Table 1.

Following a known procedure [20], the amino alcohol **5** was readily accessible from methyl glycinate hydrochloride by the reaction with excess phenylmagnesium bromide. Subsequent condensation with 1-formyl-2-naphthol (**6**) gave the imine **7** in 87% yield (Scheme 2). In order to generate the boronate–imine complex **8**, the imine **7** was treated with 4-chlorophenylboronic acid in boiling toluene in the presence of molecular sieves. The fact that this protocol led to the desired boronate complex **8** in only 17% isolated yield is attributed to steric hindrance, which originates from the diphenylhydroxymethyl group and has a disadvantageous effect on complexation. It is remarkable that considerably higher yields were obtained when regioisomeric complexes **4** were prepared via the same protocol. The formation of boronate complex **8** was indicated by its ^11^B NMR shift value of 6.6 ppm [21] and confirmed by X-ray crystal structure analysis as shown in Figure 1. Although the compound crystallized as a conglomerate, the (*S*)-enantiomer is shown arbitrarily in Figure 1a. The boron–nitrogen distance of 156.3 pm clearly shows the existence of a coordinate bond. The UV spectrum of compound **8** displays a high-wavelength absorption maximum at λ = 395 nm, typical for the imine chromophore, as observed for other boronate–imine complexes.

**Table 1:** Selected distances (Å) and angles (°) of compounds **8** and **10**·CH_3_OH.

	**8**	**10**·CH_3_OH

B1–O1	1.496(3)	1.458(6)
B1–O2	1.455(3)	1.427(5)
B1–N1	1.563(3)	1.598(6)
B1–C4	1.595(3)	1.607(6)
O1–C11	1.337(2)	1.349(5)
O2–C1	1.433(2)	1.418(5)
N1–C2	1.460(2)	1.481(5)
N1–C3	1.282(2)	1.480(5)
C1–C2	1.550(3)	1.520(5)
C3–C10	1.429(3)	1.484(6)
C10–C11	1.397(3)	1.382(6)
O1–B1–O2	111.4(2)	112.2(4)
O1–B1–N1	106.54(19)	105.9(4)
O1–B1–C4	109.59(19)	111.4(4)
O2–B1–N1	101.08(19)	101.2(4)
O2–B1–C4	113.9(2)	113.9(4)
N1–B1–C4	113.93(19)	111.5(4)
N1–H1	—	0.95(2)
H1^…^O3	—	1.99(3)
N1–H1^…^O3	—	143(3)
N1^…^O3	—	2.807(5)
O3–H2	—	0.82
H2^…^O1^a^	—	2.19
O3–H2^…^O1^a^	—	2.897(5)
O3^…^O1^a^	—	144.1

^a^−x+1, −y+2, z+0.5.

The imine **7** also served for the preparation of the boronate–amine complex **10**. For this purpose, it was reduced [22] with cyanoborohydride in methanol in the presence of hydrochloric acid to give the salt of the amine **9**. As the latter decomposed to a considerable extent when stored, it was converted immediately into the boronate–amine complex **10**. Sodium hydrogen carbonate served as the base to liberate the free amine in situ, and complexation with 4-chlorophenylboronic acid in refluxing toluene gave the complex **10** in 63% isolated yield. The lack of any absorption at wavelengths higher than 350 nm indicated the absence of the imine chromophore.

Here again the formation of the complex can be deduced from the ^11^B NMR shift of 5.5 ppm [21]. Suitable crystals of compound **10**·CH_3_OH were obtained from methanol/hexane and the result of the X-ray analysis is shown in Figure 1. A comparison of the boron–nitrogen distance in the imine complex **8** (156.3 pm) and the amine complex **10** (159.5 pm) reveals a small elongation of the bond upon saturation of the imine group that results from the change in hybridization at the nitrogen from sp^2^ to sp^3^. The structure determination **10**·CH_3_OH reveals a hydrogen bridge of the amine hydrogen atom directed to the oxygen atom of the alcohol. The fact that the NH group functions as the hydrogen-bond donor is attributed to an enhanced acidity that is caused by the coordinate bond between nitrogen and boron. Related hydrogen bonding has been observed in diastereomerically pure boronate–amine complexes [9].

Upon complexation of the boron, a typical pyramidalization occurs that can be measured in terms of the tetrahedral character (THC). For the complexes **8** and **10**, this has been calculated by taking into account the six bond angles θ_1_–θ_6_ using the equation of Höpfl (Equation 1) [23].

[1]
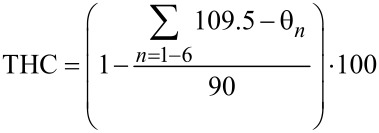


The corresponding values obtained for the boron and the nitrogen atoms in the complexes **8** and **10** are given in Table 2.

**Table 2:** Bond length and tetrahedral character (THC) of boronates **8** and **10**.

Complex	BN distance	THC boron	THC nitrogen

**8**	156.3 ppm	75%	—
**10**	159.5 ppm	75%	71%

**Figure 2:**
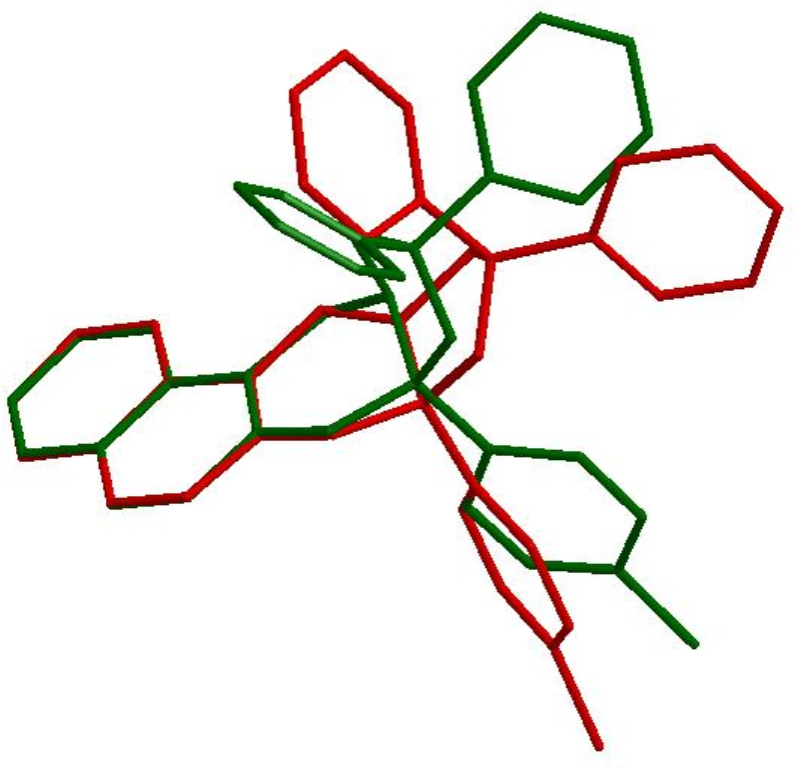
Superposition of the skeletons of boronate complexes **8** (red) and **10** (green).

Thus, the tetrahedral character is essentially identical in both complexes for the boron atom. The pyramidalization of nitrogen that occurs upon saturation of the imine bond is evident from the values of the tetrahedral character of the nitrogen atom in the amine complex. The saturation of the carbon–nitrogen bond has a considerable impact on the structure of both of the complexes **8** and **10**. This becomes evident from Figure 2, which shows a superposition of the skeletons of complexes **8** (red) and **10** (green). As a consequence of the saturation and the rehybridization at nitrogen, the five-membered rings are connected in a more concave, folded manner in the amine complex **10** than in the rather flat junction of both rings in the imine complex **8**. It is possible that steric repulsion is more severe in the amine complex **10** that could slightly weaken the boron–nitrogen bond.

In order to prove the enantiomerism based upon stereogenic boron, the racemates of the complexes **8** and **10** were resolved by HPLC on a chiral column. Both complexes displayed peaks of identical intensity resulting from the enantiomers. The differences in the retention times permitted isolation of the pure enantiomers on a semi-preparative scale. Mirror-image CD spectra were obtained for the separated enantiomers of the imine complex **8**. Previously, we have been able to assign the absolute configuration to the complex **4a**, a regioisomer of **8**, by comparison of the measured and calculated CD spectra [18]. The (*R*)-enantiomer of **4a** is characterized by an intense, positive Cotton effect at high wavelength. As a similar effect is observed in the CD spectrum of the enantiomer of **8** that is eluted at lower retention time, the (*R*)-configuration is attributed to this enantiomer by analogy. As we were interested in whether or not the configurational stability differs in boronate–imine complexes **4** and **8** on the one hand and boronate–amine complex **10** on the other hand, the racemization barrier of the latter was determined. For this purpose, a sample of the enantiomerically pure complex **10** was heated in *n*-decane at 60 °C, and the decay of the optical purity was followed by chiral HPLC. Thus, the racemization rate at 325.4 K was measured to be 4.1·10^−4^ s^−1^ and the racemization barrier ΔG^‡^ amounted to 101.0 kJ·mol^−1^ [24]. The corresponding values for boronate–imine complexes **4** varied from 105–110 kJ·mol^−1^, indicating that the change from the imine to the amine ligand does not alter the configurational stability at boron to any considerable degree. The racemization process involves the breaking of the coordinate boron–nitrogen bond to produce a trigonal boron atom that is then attacked by the nitrogen from the opposite face. As the nitrogen–boron distances were similar in all the boronate complexes **4**, **8**, and **10**, it is plausible that the racemization barrier is also in the same range for all of these compounds.

## Conclusion

In summary, it was shown that boron based enantiomerism is not only possible in boronate–imine complexes **4** and **8**, but also in amine complexes as well as exemplified by complex **10** that differs from **8** in the saturation of the imine bond to an amine bond. Enantiomers of boronate complexes **8** and **10** are accessible and were shown to be stable at room temperature. The racemization barriers are of similar magnitude in the amine and imine complexes.

## Experimental

General: Melting points (uncorrected) were determined with a Büchi 540 melting point apparatus. NMR spectra were recorded with a Bruker DXR 500 spectrometer. Mass spectra were recorded on an ion-trap API mass spectrometer Finnigan LCQ Deca (ESI), triple-quadrupole-mass spectrometer Finnigan TSQ 7000, and sector field mass spectrometer Finnigan MAT 8200 (EI, 70 eV). High resolution mass spectra were carried out on Bruker FT-ICR APEX III (7.0 T) (MALDI) at the University of Bielefeld. Column chromatography was performed with Fluka silica gel 60 (230–400 mesh) and thin layer chromatography was carried out on Merck TLC Silicagel 60 F_254_ aluminium sheets. HPLC was performed with the chiral column CHIRALPAK IB. Toluene was freshly distilled from sodium under a nitrogen atmosphere.

### 

#### 1-{[(2-Hydroxy-2,2-diphenylethyl)imino]methyl}naphthalen-2-ol (7)

A mixture of amino alcohol **5** (0.427 g, 2.00 mmol), naphthaldehyde **6** (0.379 g, 2.20 mmol), and sodium sulfate (2.0 g, 14 mmol) was suspended in absolute methanol (25 mL) and dry toluene (25 mL) under a nitrogen atmosphere. The yellow mixture was heated under reflux with stirring for 18 h. After cooling to room temperature, the sodium sulfate was removed by filtration and the filtrate concentrated in a rotary evaporator. The residue was purified by column chromatography (chloroform/ethyl acetate, 10:1) to give product **7** as a solid (0.640 g, 87%). *R*_f_ 0.1 (chloroform/ethyl acetate, 10:1); mp 178 °C; ^1^H NMR (500 MHz, CDCl_3_) δ 1.90 (s, 1H, aliphatic OH), 3.30 (d, *J* = 2.6 Hz, 2H, CH_2_), 6.90 (d, *J* = 9.3 Hz, 1H, 3-H), 7.18 (m, 1H, 6-H), 7.2–7.3 (m, 10H, phenyl H), 7.36 (t, *J* = 7.0 Hz, 1H, 7-H), 7.57 (d, *J* = 7.9 Hz, 1H, 5-H), 7.66 (d, *J* = 9.3 Hz, 1H, 4-H), 7.74 (d, *J* = 8.4 Hz, 1H, 8-H), 8.57 (d, *J* = 6.7 Hz, 1H, N=CH), 14.8 (broad s, 1H, phenolic OH); ^13^C NMR (125 MHz, CDCl_3_) δ 59.9 (CH_2_), 73.8 (CPh_2_), 107.0 (C-1), 118.1 (C-8), 122.9 (C-6), 123.9 (C-3), 126.3 (C-9), 126.9–130.6 (phenyl C), 129.5 (C-5), 133.3 (C-10), 136.0 (phenyl ipso-C), 137.0 (C-4), 160.2 (C-11), 174.2 (C-2); MS (ESI) *m/z* (%): 368 [M + H]^+^ (40), 350 (100), 196 (30).

#### Boronate–imine complex 8

The imine **7**, (0.367 g, 1.00 mmol), 4-chlorophenylboronic acid (0.183 g, 1.50 mmol) and 1.0 g of molecular sieves (3 Å) were suspended in 100 mL of dry toluene and heated under reflux for 20 h. After filtration, the solvent was removed in a rotary evaporator and the residue purified by column chromatography (chloroform/ethyl acetate, 10:1) to afford boronate **8** as a yellow solid (80 mg, 17%). *R*_f_ 0.76 (chloroform/ethyl acetate, 10:1); mp 126 °C; ^1^H NMR (500 MHz, CDCl_3_) δ 4.52 (d, *J* = 11.0 Hz, 1H, C*H*H), 4.61 (d, *J* = 11.0 Hz, 1H, CH*H*), 6.99 (t, *J* = 7.4 Hz, 1H, phenyl H), 7.03 (d, *J* = 7.4 Hz, 2H, *o*-chloro H), 7.1–7.54 (m, 9H, phenyl H), 7.17 (d, *J* = 9.0 Hz, 1H, naphthyl 3-H), 7.29 (d, *J* = 7.4 Hz, 2H, *m*-chloro H), 7.31 (m, 1H, naphthyl 6-H); 7.42 (d, *J* = 8.0 Hz, 1H, naphthyl 7-H), 7.61 (d, *J* = 8.0 Hz, 1H, naphthyl 8-H), 7.65 (d, *J* = 8.1 Hz, 1H, naphthyl 5-H), 7.85 (d, *J* = 9.1 Hz, 1H, naphthyl 4-H), 8.5 (s, 1H, N=CH); ^13^C NMR (125 MHz, CDCl_3_) δ 65.1 (CH_2_), 82.4 (CPh_2_), 109.8 (naphthyl C-1), 119.7 (naphthyl C-8), 121.2 (naphthyl C-3), 125.8–128.9 (phenyl C), 126.1 (*o*-chloro C), 127.5 (naphthyl C-9), 129.3 (naphthyl C-5), 131.6 (naphthyl C-10), 133.1 (*m*-chloro C), 139.2 (naphthyl C-4), 145.2 (phenyl ipso-C), 146.1 (phenyl ipso-C), 154.1 (N=CH), 162.7 (naphthyl C-2); ^11^B NMR (160 MHz, CDCl_3_) δ 6.6; MS (ESI) *m/z* (%): 510 [M + Na]^+^ (10), 489 [M + H]^+^ (20), 376 (35) 368 (100); HRMS calcd for C_31_H_23_NO_2_BCl, [M + Na]^+^ 509.14389; found, 509.14438, [M + H]^+^ 487.16195; found, 487.16219.

#### Boronate–amine complex 10

The imine **7**, (0.367 g, 1.00 mmol) and sodium cyanoborohydride (0.19 g, 3.0 mmol) were dissolved in absolute methanol (75 mL). After adding 5 mL of hydrochloric acid (10%), the yellow solution gradually became colorless on stirring at room temperature for 1 h. Distilled water (50 mL) was added and the solution extracted three times with chloroform. The combined organic layers were dried with sodium sulfate and the solvent was removed in a rotary evaporator. Immediately, the resulting crude colorless product, 4-chlorophenyl boronic acid (0.122 g, 1.00 mmol) and sodium hydrogen carbonate (0.13 g, 1.5 mmol) were suspended in 100 mL of dry toluene and heated under reflux for 4 h. After the addition of distilled water, the layers were separated and the aqueous phase was extracted with chloroform. The combined organic layers were dried with sodium sulfate and the solvent was removed in a rotary evaporator. The residue was purified by column chromatography (chloroform/ethyl acetate, 10:1) to yield boronate **10** as a colorless solid (0.210 g, 63%). *R*_f_ 0.76 (chloroform/ethyl acetate, 10:1), mp 157 °C; ^1^H NMR (500 MHz, CDCl_3_) δ 3.53 (t, *J* = 11.5 Hz, 1H, Ph_2_C–C*H*H), 3.83 (dd, *J* = 11.4 Hz, *J* = 5.5 Hz, 1H, Ph_2_C–CH*H*), 3.95 (dd, *J* = 15.3 Hz, *J* = 4.3 Hz, 1H, napththyl C*H*HN), 4.30 (d, *J* = 15.4 Hz, 1H, naphthyl CH*H*N), 4.34 (quint, *J* = 5.6 Hz, 1H, NH), 6.92 (d, *J* = 9.0 Hz, 1H, naphthyl 3-H), 6.94 (d, 2H, *o*-chloro H), 7.0–7.25 (m, 10H, phenyl H), 7.23 (d, *J* = 8.2 Hz, 2H, *m*-chloro H), 7.29 (t, *J* = 8.3 Hz, 1H, naphthyl 8-H), 7.54 (d, *J* = 9.2 Hz, 1H, naphthyl 4-H), 7.64 (d, *J* = 8.0 Hz, 1H, naphthyl 5-H); ^13^C NMR (125 MHz, CDCl_3_) δ 43.6 (naphthyl CH_2_), 58.5 (Ph_2_C-*C*H_2_), 82.0 (Ph_2_C), 104.6 (naphthyl C-1), 120.0 (naphthyl C-8), 121.5 (naphthyl C-3), 122.7 (naphthyl C-6), 125.8–128.5 (phenyl C), 128.0 (*o*-chloro C), 128.1 (naphthyl C-9), 128.5 (naphthyl C-5), 130.0 (naphthyl C-4), 131.6 (naphthyl C-10), 133.8 (*m*-chloro C), 145.7 (phenyl ipso-C), 146.4 (phenyl ipso-C), 152.9 naphthyl C-2); ^11^B NMR (160 MHz, CDCl_3_) δ 5.5; MS (ESI) *m/z* (%): 512 [M + Na]^+^ (97), 490 [M + H]^+^ (32), 370 (32), 196 (100); HRMS calcd for C_31_H_25_NO_2_BCl, [M + K]^+^ 527.13348; found, 527.13353; [M + Na]^+^ 511.15954, found, 511.15935.

#### Crystal structure determination

Crystals of compounds **8** and **10**·CH_3_OH suitable for X-ray analysis were selected by means of a polarization microscope and investigated with a STOE imaging plate diffraction system using graphite monochromatic MoKα radiation (λ = 0.71073 Å). To avoid loss of methanol, and deterioration, crystals of compound **10**·CH_3_OH were enclosed in thin walled glass capillaries. Unit cell parameters were determined by least-squares refinements on the positions of 8000 and 3769 reflections in the range 2.25° < θ < 25.25° and 1.95° < θ < 19.70°, respectively. Space group type no. 61 was uniquely determined in the case of compound **8**. For crystals of compound **10**·CH_3_OH Laue class 4/*m* and serial systematic extinctions were consistent with the enantiomorphic pair *P*4_1_–*P*4_3_. In the course of refinement *P*4_1_ was shown to be the correct choice for the crystal under investigation. Data sets were corrected for Lorentz and polarization effects in both cases. Crystal data, as well as details of data collection and structure refinement are listed in Table 3.

**Table 3:** Summary of crystal data, details of intensity measurements and structure refinements of **8** and **10**·CH_3_OH.

	**8**	**10**·CH_3_OH

Empirical formula	C_31_H_23_BClNO_2_	C_32_H_29_BClNO_3_
M_r_	487.76	521.82
Crystal system	orthorhombic	tetragonal
Space group	*Pbca*	*P*4_1_
Z	8	4
Temperature [K]	291(2)	291(2)
Unit cell parameters		
*a* [Å]	10.7877(6)	15.1310(11)
*b* [Å]	18.0573(11)	
*c* [Å]	25.9072(13)	11.8802(8)
Volume [Å^3^]	5046.6(5)	2719.9(5)
*D*_calcd_	1.284	1.274
Absorption coefficient	0.181	0.175
*F*(000)	2032	1096
Crystal size [mm^3^]	0.12 × 0.12 × 0.12	0.17 × 0.13 × 0.13
Crystal color	yellow	colorless
Diffractometer type	Stoe-IPDS	Stoe-IPDS
Scan mode	φ	φ
θ range for data collection	2.26-25.00	2.18-24.99
Limiting indices	−12<*h*<12	−17<*h*<17
	−21<*k*<21	−17<*k*<17
	−30<*l*<30	−13<*l*<13
Reflections collected	62403	30038
Reflections unique	4441	4761
Reflections observed	1731	1274
Criterion for observation	*I*>2σ(*I*)	*I*>2σ(*I*)
Completeness	1.000	0.996
Refined Parameters	325	347
*R*_1_^a^, observed	0.036	0.035
*wR*_2_^b^, all data	0.070	0.050
Goodness-of-Fit, *S*^c^	0.90	0.82
x(Flack)		−0.03(8)
Largest diff. peak/hole	0.26/−0.17	0.10/−0.09
CCDC-identifier	809030	809031

^a^*R*_1_ = || *F*_o_|-|*F*_c_||/|*F*_o_|; ^b^*wR*_2_ = [*w*(*F*_o_^2^-*F*_c_^2^)^2^/*w*(*F*_o_^2^)^2^]^1/2^, where *w* = 1/[*σ*^2^(*F*_o_^2^)+(a*P*^2^)] and *P* = (*F*_o_^2^+2*F*_c_^2^)/3; ^c^*S* = [*w*(*F*_o_^2^-*F*_c_^2^)^2^/(n-p)]^1/2^.

The structures were solved by direct methods [25] and subsequent Δ*F*-syntheses and approximate positions of all the hydrogen atoms were found. Anisotropic displacement parameters were applied for all atoms heavier than hydrogen, and, using the subsequently mentioned restraints and constraints, refinements [26] by full-matrix least-squares calculations on *F*^2^ converged (max. shift/esd: 0.000 and 0.001, respectively). For the NH group of **10**·CH_3_OH, weak N–H bond length restraints were applied. With bond lengths and angles constrained to idealized values for the CH_3_, CH_2_, CH, and OH groups of the boron complexes and for the methanol molecule of **10**·CH_3_OH, the riding model was applied for all the other H atoms. In addition, the H atoms of the CH_3_ group of the methanol molecule were allowed to move collectively around the neighbouring C–C axis, and the H atom of the OH group was also allowed to rotate around the neighbouring O–C axis. Isotropic displacement parameters of H atoms were constrained to 120% of the equivalent isotropic displacement parameters of the parent N and C atoms for the NH, CH and CH_2_ groups and to 150% of the parent C and O atoms for the CH_3_ group and the OH group of the methanol molecule in **10**·CH_3_OH. CCDC-809030 (compound **8**) and CCDC-809031 (compound **10**·CH_3_OH) contain the supplementary crystallographic data (excluding structure factors) for this paper. These data can be obtained free of charge from The Cambridge Crystallographic Data Centre via http://www.ccdc.cam.ac.uk/data_request/cif.
